# Disequilibrium of Flavonol Synthase and Dihydroflavonol-4-Reductase Expression Associated Tightly to White vs. Red Color Flower Formation in Plants

**DOI:** 10.3389/fpls.2015.01257

**Published:** 2016-01-13

**Authors:** Ping Luo, Guogui Ning, Zhen Wang, Yuxiao Shen, Huanan Jin, Penghui Li, Shasha Huang, Jian Zhao, Manzhu Bao

**Affiliations:** ^1^Key Laboratory of Horticultural Plant Biology, Ministry of Education, College of Horticulture and Forestry Sciences, Huazhong Agricultural UniversityWuhan, China; ^2^College of Plant Science and Technology, Huazhong Agricultural UniversityWuhan, China

**Keywords:** anthocyanins, flavonols, flower color, disequilibrium, *FLS*, *DFR*

## Abstract

Flower color is the main character throughout the plant kingdom. Though substantial information exists regarding the structural and regulatory genes involved in anthocyanin and flavonol biosynthesis, little is known that what make a diverse white vs. red color flower in natural species. Here, the contents of pigments in seven species from varied phylogenetic location in plants with red and white flowers were determined. Flavonols could be detected in red and white flowers, but anthocyanins were almost undetectable in the white cultivar. Comparisons of expression patterns of gene related to the flavonoid biosynthesis indicated that disequilibrium expression of flavonol synthase (*FLS*) and dihydroflavonol-4-reductase (*DFR*) genes determined the accumulation of flavonols and anothcyanins in both red and white flowers of seven species. To further investigate the role of such common regulatory patterns in determining flower color, *FLS* genes were isolated from *Rosa rugosa* (*RrFLS1*), *Prunus persica* (*PpFLS*), and *Petunia hybrida* (*PhFLS*), and *DFR* genes were isolated from *Rosa rugosa* (*RrDFR1*) and *Petunia hybrida* (*PhDFR*). Heterologous expression of the *FLS* genes within tobacco host plants demonstrated conservation of function, with the transgenes promoting flavonol biosynthesis and inhibiting anthocyanin accumulation, so resulting in white flowers. Conversely, overexpression of *DFR* genes in tobacco displayed down-regulation of the endogenous *NtFLS* gene, and the promotion of anthocyanin synthesis. On this basis, we propose a model in which FLS and DFR gene-products compete for common substrates in order to direct the biosynthesis of flavonols and anthocyanins, respectively, thereby determining white vs. red coloration of flowers.

## Introduction

Flavonoids are major secondary metabolites in plants including the flavonens, isoflavones, anthocyanins, flavones, and proanthocyanidins that provide plants with varied pigments (Dixon and Steele, 1999; Winkel-Shirley, 2001; Grotewold, 2006; Tanaka et al., 2008). Flower color is clearly an important horticultural trait within ornamental plants, and pigmentation is also a major factor in attracting pollinators (Shang et al., 2011; Shrestha et al., 2013). Of which, anthocyanins are the most common pigments found in flowers and fruits (Tanaka et al., 2008; Morita et al., 2014) and, thus, are of particular importance.

**Figure 1:**
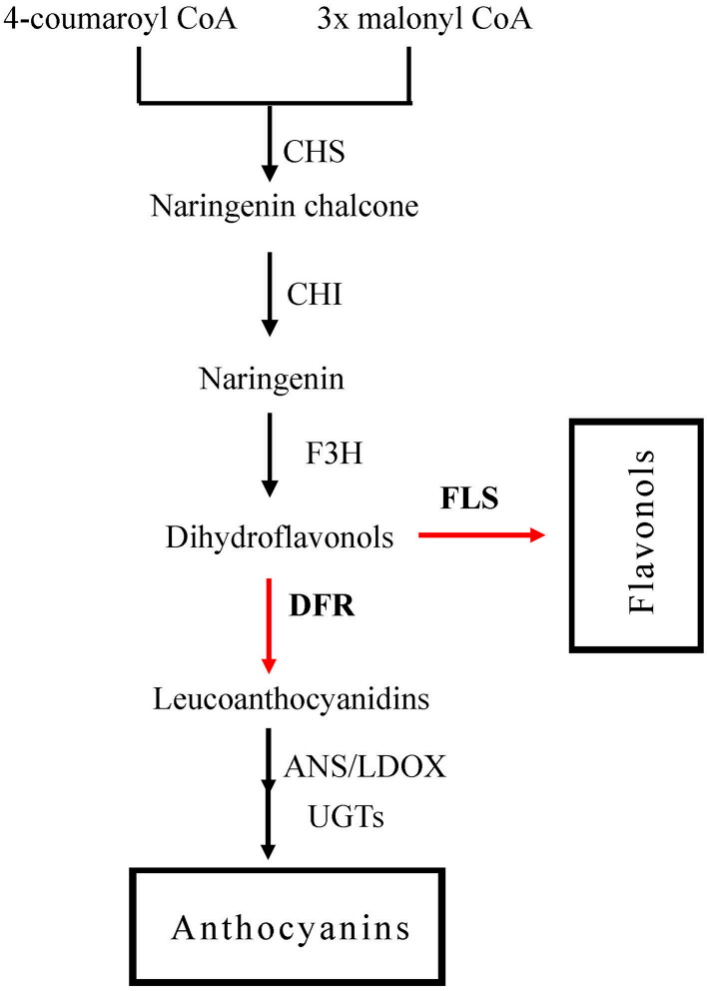
**The flavonoid pathway leading to anthocyanins and flavonols**. Enzymes are as follows: CHS, chalcone synthase; CHI, chalcone isomerase; F3H, flavonoid 3-hydroxylase; FLS, flavonol synthase; DFR, dihydroflavonol reductase; ANS, anthocyanidin synthase; LDOX, leucoanthocyanidin di-oxygenase; UGTS, glucose transferase.

Two major components, namely dihydrokaempferol and dihydroquercetin could be catalyzed either by FLS to form a variety of copigment flavonols and glycosidic derivatives (Li et al., 2012), or by DFR in the anthocyanin biosynthesis pathway (Figure 1; Harborne and Williams, 2000). The secondary metabolic pathway leading to the anthocyanins biosynthesis has been widely studied in several plant species (Jaakola et al., 2002; Saito and Yamazaki, 2002; Shih et al., 2006; Ring et al., 2013). Dihydroflavonol-4-reductase (DFR) controls one of the rate-limiting steps in the pathway and catalyzes the stereospecific reduction of three dihydroflavonols to leucoanthocyanidins (Martens et al., 2003; Shimada et al., 2005; Lo-Piero et al., 2006). This critical role of DFR in the flavonoid pathway is reflected in the focus of research programmes, and *DFR* gene homologs have been isolated from numerous plant species including *Medicago truncatula* (Xie et al., 2004), *Lotus japonicas* (Shimada et al., 2005), *Camellia sinensis* (Singh et al., 2009), and *Populus trichocarpa* (Huang et al., 2012).

Flavonols represent another key subgroup of flavonoids which play pivotal roles in the pigmentation of flowers (Czemmel et al., 2009). The flavonol derivatives have been shown to influence anthocyanin-mediated coloration by copigmentation effects. Flavonol synthase (FLS) catalyzes the conversion of dihydroflavonols to flavonol, and thus may influence levels of anthocyanin accumulation (Owens et al., 2008; Kuhn et al., 2011). The first *FLS* gene was identified from *P. hybrida* (Holton et al., 1993). Subsequently, other *FLS* genes were cloned from varied plant species, including *A. thaliana* (Pelletier et al., 1999; Owens et al., 2008), *E. grandiflorum* (Nielsen et al., 2002), and *G. max* (Takahashi et al., 2007). In addition, it has been found that the enhanced the expression level of *FtFLS1* and *FtFLS2* from tartary buckwheat correlated with higher content of flavonols (Li et al., 2013). Recently, it has been reported that overexpression of *BnFLS* from *Brassica napus* in *Arabidopsis* atfls1-ko mutant, induced the accumulation of flavonol (Vu et al., 2015).

Substantial information exists regarding the structural and regulatory genes involved in anthocyanin and flavonol biosynthesis. However, it still unclear that how the structural genes cooperate to control the metabolic balance between the anthocyanin and flavonol branches of the flavonoid pathway, and that what make a diverse white vs. red color flower in many natural species. In this study, we firstly investigated the expression of flavonoid biosynthesis genes related to the accumulation of anthocyanins and flavonols within red flower and white flower phenotypes of the roses *Rosa rugosa* and *Rosa multiflora*, peach (*Prunus persica*), carnation (*Dianthus caryophyllus*), azalea (*Rhododendron simsii*), camellia (*Camellia japonica*), and *petunia* (*Petunia hybrida*). We also isolated clones of *FLS* and *DFR* homologs from these species and checked for functionality of the genes using ectopic expression systems in tobacco. Overexpression of *FLS* in tobacco flowers was found to inhibit anthocyanin biosynthesis and promote flavonol synthesis, and this was reflected in the accumulated levels of the respective metabolites. In contrast, overexpression of the various *DFR* genes in tobacco flowers promoted anthocyanin biosynthesis, corresponding to an increase in red pigmentation. These findings are consistent with the relationship between the expression profiles of *DFR* and *FLS* and the accumulations of anthocyanin and flavonol in natural species with white vs. red color flower. Thus, we conclude that *FLS* and *DFR* enzymes direct the biosynthesis of flavonols and anthocyanins, respectively, thereby directing the development of white vs. red color flower phenotypes in plant.

## Materials and methods

### Plant materials and growing conditions

The red and white flower phenotypes of the roses *Rosa rugosa* and *Rosa multiflora*, peach (*Prunus persica*), carnation (*Dianthus caryophyllus*), azalea (*Rhododendron simsii*), camellia (*Camellia japonica*), and *petunia* (*Petunia hybrida*), as used for gene cloning, quantitative PCR and metabolite analyses, were field-grown in the experimental plots of Huazhong Agricultural University, Wuhan, China. The early-flowering tobacco variety described by Ning et al. (2012) was used for genetic transformation experiments. The transgenic tobacco lines were cultured in a growth chamber with a 25° C/17°C day/night temperature regime under 14 h light/10 h dark photoperiod. Flowers were harvested in full bloom for gene expression and pigment measurement. All samples were frozen in liquid nitrogen and stored at −80°C until analysis.

### Expression analysis by quantitative real-time PCR (qRT-PCR)

Total RNA was isolated with CTAB as described previously (Hu et al., 2002). cDNA synthesis was performed with PrimeScript™ RT-PCR Kit (Takara, Japan). qRT-PCR was conducted with the SYBR Premix Ex Taq (Takara, Japan) and ABI7500 cycler (Applied Biosystems, Foster City, CA, USA). The PCR amplification program was described by Xing et al. (2014). The 2[-Delta Delta C(T)] was applied to the determination of transcript levels. 18S or EF1α was used as house-keeping gene to normalize the relative expression level of the analyzed genes in camellia or tobacco, respectively. GAPDH was used as an internal control in rose and carnation. Actin was used to obtain the normalized expression of the target gene in petunia and peach. All analyses were performed with three biological replicates. The specific primers sequence (Table S3) for qRT-PCR were designed with primer 5 program.

### Construction of *RrDFR1, PhDFR, RrFLS1, PhFLS1*, and *PpFLS* plant overexpression vectors and tobacco transformation procedures

The RrDFR1qF and RrDFR1qR primers (containing *Bam*HI and *Sal*I, respectively), were designed to amplify the full-length *RrDFR1* ORF (GenBank accession no. KM203111). The full-length *PhDFR* ORF (GenBank accession no. AF233639.1) was amplified with PhDFRqF and PhDFRqR primers containing the restriction sites *Kpn*I and *Bam*HI, respectively. The complete coding sequence of the *RrFLS1* (GenBank accession no. KM099095) gene was obtained using the primer pairs RrFLS1qF and RrFLS1qR. The complete coding sequences of *PhFLS* (GenBank accession no. Z22543.1) and *PpFLS* (GenBank accession no. KP050782) were obtained from GenBank. The full-length *PhFLS* ORF and *PpFLS* ORF were amplifled by RT-PCR, and contained the restriction sites *Bam*HI and *Sal*I. All primers sequence used for gene cloning are shown in Table S2. The PCR was performed using *Pfu* DNA Polymerase (Stratagene, La Jolla, CA). The amplified products were cloned into the pMD18-T vector (Takara, Japan), and the identity confirmed by sequencing (Sangon, Shanghai, China). After digestion with the appropriate restriction enzymes, the inserts were ligated into the modified binary vector pCAMBI2300S (Figure S2). The amino acid secquence alignment was performed using ClustalW program and the Neighbor–Joining (NJ) method was used to construct the phylogenetic tree by MEGA 4 software.

Each construct was transformed into *Agrobacterium tumefaciens* strain EHA105 by electroporation. Agrobacterium-mediated tobacco transformation was conducted according to our privous study (Ning et al., 2012).

### Measurement of flavonol and anthocyanin contents

Petals were harvested from the fully open flowers and were homogenized in extraction solution (0.1% HCl in methanol) followed by incubation at 4°C in the dark for 24 h. The supernatants was transferred to a clean tube and filtered through a 0.2 μm Tefion filter before analysis. The extract was analyzed using SHIMADZU HPLC with TSK ODS-80Ts QA (4.6 mm × 250 mm, 5 μm, Tosoh) column. The volume of injection was 10 μl. Milli-Q water containing 10% (v:v) formic acid was used as solution A and CH_3_CN containing 0.1% (v:v) formic acid was used as solution B. The linear elution gradient method was as follows: 0–20 min, 10% B; 20–25 min, 30% B; 25–35 min, 10% B. at 520 and 360 nm for anthocyanins and flavonols, respectively. The quantification of the anthocyanins was achieved as cyanidin Chromatograms were acquired 3-O-glucoside (Sigma, USA) equivalents. The flavonol level was determined with quercetin-3-O-rutinoside (Sigma, USA) equivalents (Bogs et al., 2005). For the analysis of the anthocyanin content of tobacco flowers, the samples were extracted with acidic MeOH in the dark for 48 h. The level of anthocyanins was determined according to Zhang et al. (2009): Q_Anthocyanins_ = (A_530_-0.25 × A_657_) × M^−1^. All analyses were performed with three biological replicates.

### Statistical analysis

The analysis of variance (ANOVA) were performed using SAS program (version 8.0, SAS Institute, NC, USA). The statistical difference was compared by the Fisher's LSD test (*P* < 0.05 as significant).

## Results

### Anthocyanin and flavonol metabolites accumulate differentially in red and white flowers

**Figure 2:**
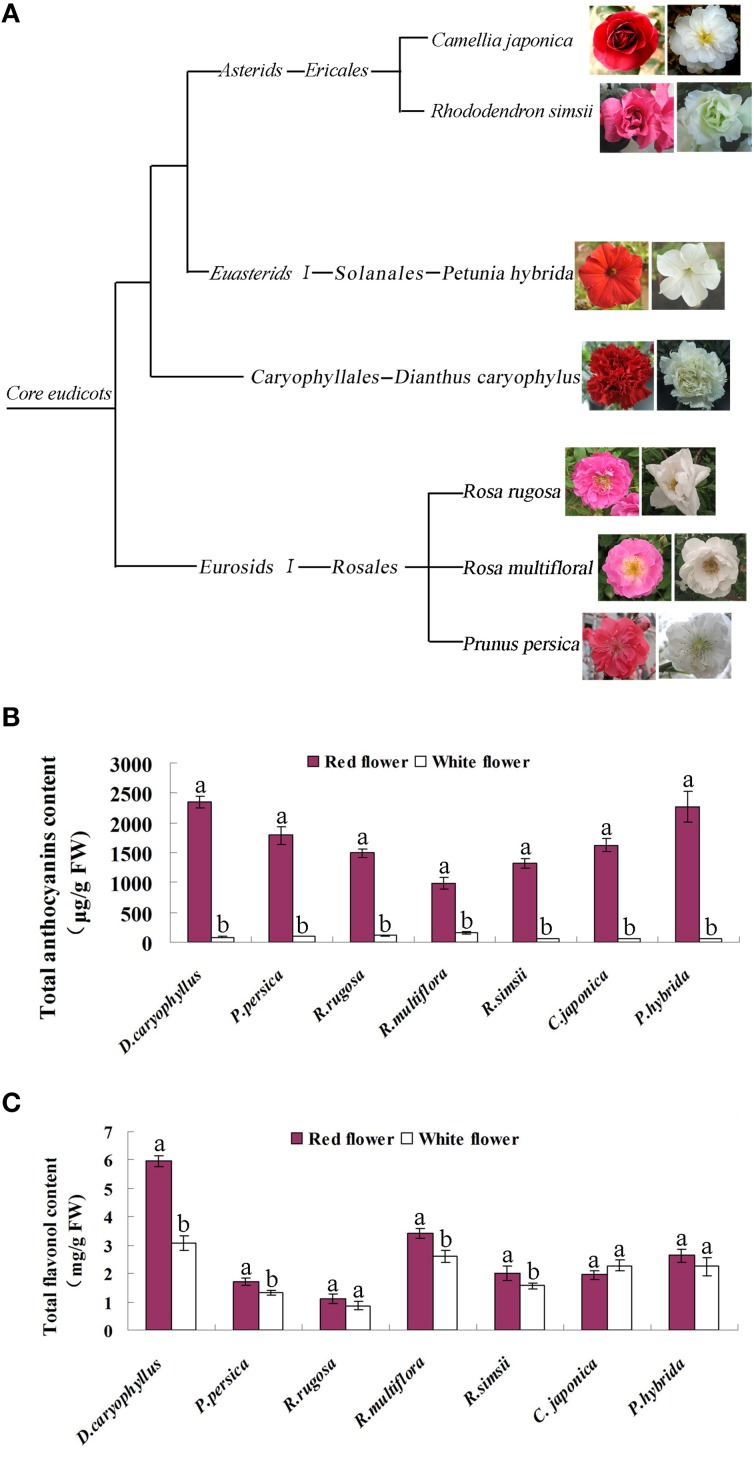
**Characterization of the seven plant species used in this study**. **(A)** The phylogenetic classification of the seven species. **(B)** Levels of anthocyanin in the petals of the red flower and white flower phenotypes of each species. **(C)** Levels of flavonol in the petals of the red flower and white flower phenotypes of each species. Different letters above the error bar indicate that values of the red flowers were significantly different from those of white flowers at *P* < 0.05 using the Fisher's LSD test.

We extracted metabolites from seven species of plants possessing red and white flower forms, namely, the roses *Rosa rugosa* and *Rosa multiflora*, peach (*Prunus persica*), carnation (*Dianthus caryophyllus*), azalea (*Rhododendron simsii*), camellia (*Camellia japonica*), and *petunia* (*Petunia hybrida*) (Figure 2A). HPLC analysis revealed that the flavonol content of red and white flowers in the respective species did not consistently show a significant difference (Figure 2C). By contrast, the anthocyanin content of the red flowers in all 7 species was found to be significantly higher than that in the white flowers. For example, in the rose and peach species levels of anthocyanin in the red flowers were increased 13.3- and 18.3-fold, respectively, compared to the levels found in the corresponding white flowers. Similarly, the anthocyanin content of red azalea flowers was 26.5-fold higher than that in the white flowers (Figure 2B). In white flowers, flavonols formed the dominant pigment type, with anthocyanin levels being substantially lower. Thus, the flavonol content of white flowers of carnation was measured as 3.08 mg/g FW whereas, the anthocyanin content in white flowers was 80.25 μg/g FW. Clearly, therefore, white and red flowers were characterized by significantly different levels of anthocyanin accumulation and this also corresponded to marked differences in the ratio of anthocyanin and flavonol pigments in the two flower colors.

### Flavonoid pathway analysis implicates differential gene expression in red and white flowers

**Figure 3:**
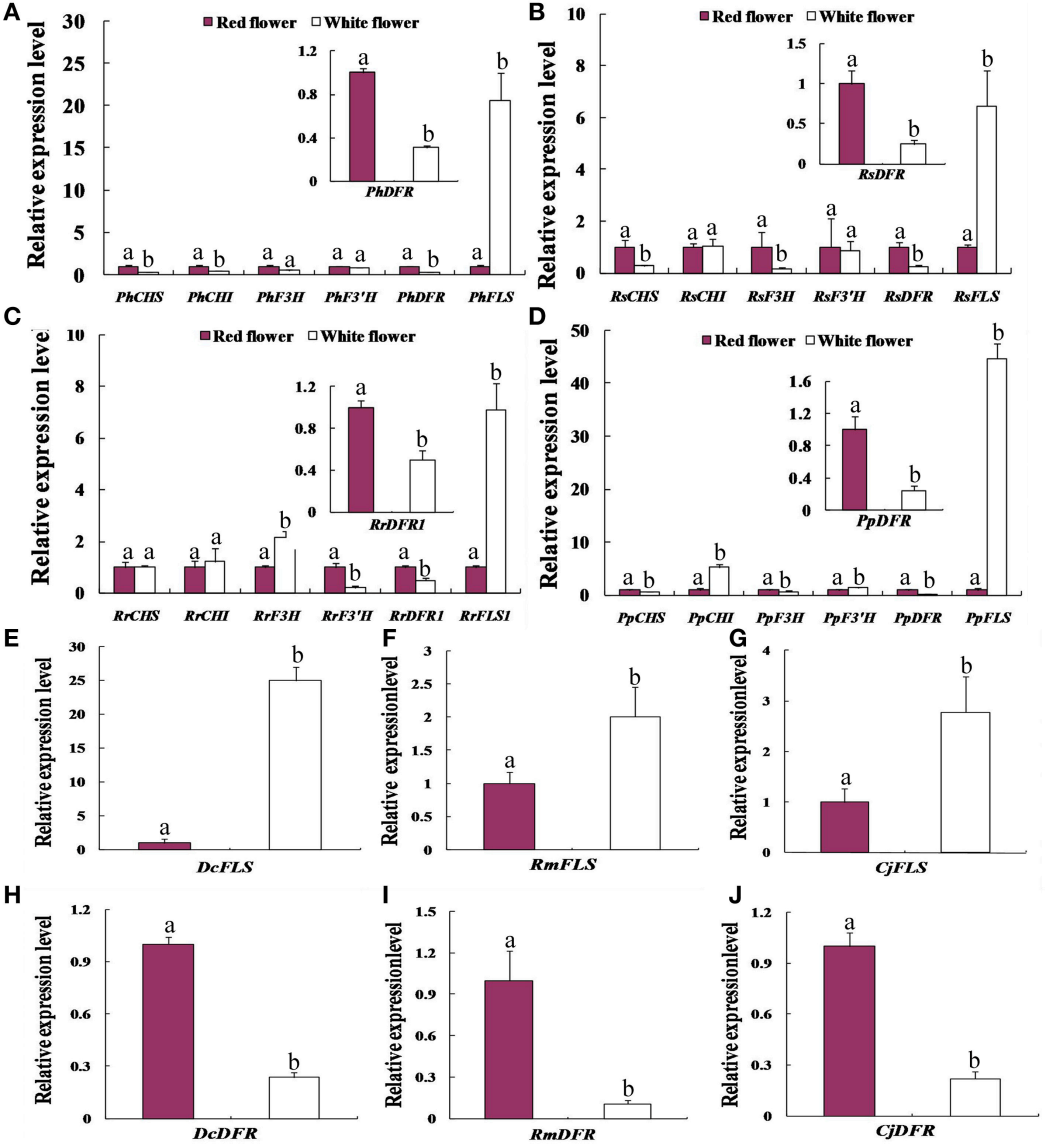
**Comparative expression patterns of genes encoding key enzymes of flavonol and anthocyanin biosynthesis in the red flowers and white flowers of the seven plant species**. **(A)** *Petunia hybrida*. **(B)** *Rhododendron simsii*.**(C)** *Rosa rugosa*. **(D)** *Prunus persica*. **(E,H)** the expression level of *DFR* and *FLS* in *Dianthus caryophyllus*. **(F,I)** the expression level of *DFR* and *FLS in Rosa multiflora*. **(G,J)** the expression level of *DFR* and *FLS in Camellia japonica*. Enzyme names are abbreviated as in Figure 1. Different letters above the error bar indicate significantly different values (*P* < 0.05) using the Fisher's LSD test.

We compared the expression profiles of key genes related to flavonoid synthesis in red and white flowers, using real-time PCR. This analysis (Figure 3) indicated that the transcript levels of chalcone isomerase (*CHI*), chalcone synthase (*CHS*), and flavonoid 3-hydroxylase (*F3H, F3*′*H)* genes showed only minor differences between the red and white flower phenotypes of *P. hybrida, R. simsii, R. rugosa*, and *P. persica*. By contrast, the transcript levels of *DFR* and *FLS* genes in these four species showed significantly different expression levels in the two flower colors (Figure 3). Thus, in *Petunia hybrida, FLS* transcript levels were found to be 20.6-fold higher in white flowers compared to red flowers whereas, *DFR* transcript levels were 3.2-fold higher in red flowers than in white flowers (Figure 3A). The expression levels of *FLS* in the white flowers of peach were as much as 44.6-fold higher than in red flowers (Figure 3D). Similarly, *RrFLS1* in the white flowers of *R. rugosa* showed a 7.1-fold increase in expression compared to that in red flowers (Figure 3C) whereas, the expression of *RrDFR1* in white flowers was 2-fold lower than that in red flowers (Figure 3C). In white flowers of *R. simsii*, expression of *RsFLS* was 6.4-fold higher than in the red flowers (Figure 3B), and *RsDFR* was 4.1-fold higher in red, as compared to white flowers (Figure 3B). These results across the four plant species, therefore, consistently demonstrated a significant difference in the expression patterns of *DFR* and *FLS* genes between the white and red flower colors.

In order to test further whether such differential expression levels of *FLS* and *DFR* genes are common to the white vs. red flower phenotypes, we analyzed three further plant species namely, *D. caryophyllus, C. japonica*, and *R. multiflora*. The results of real-time PCR analyses indicated that the transcript abundances detected in the white flowers, relative to the respective red flower forms, were 25-fold higher for *DcFLS* (Figure 3E), 2.8-fold higher for *CjFLS* (Figure 3G), and 2.0-fold higher for *RmFLS* (Figure 3F). By contrast, expression levels in the red flowers compared to those in the white flowers were 4.2-fold higher for the *DcDFR* gene (Figure 3H), while *CjDFR* levels were 9.3-fold higher (Figure 3J) and expression of *RmDFR* was 4.5-fold higher (Figure 3I). These results therefore support the conclusion that the different expression ratios of *DFR* and *FLS* genes between the red and white flower phenotypes are intrinsic to the formation of the two pigmented forms.

### Sequence and phylogeny analysis of FLS and DFR genes

Multiple sequence alignment of the deduced amino acids of the rose, peach and petunia *FLS* genes, *RrFLS1, PpFLS*, and *PhFLS*, was carried out against the FLS sequences from five other plant species. The predicted products of *RrFLS1, PpFLS*, and *PhFLS* were found to share high sequence identity with those of the other known *FLS* genes, and this was particularly evident across the middle portion of each sequence (Figure S1). Sequence analysis showed that the predicted RrFLS1, PpFLS, and PhFLS sequences contained a highly conserved N-terminal domain typical of the 2OG-Fe(II) dioxygenase and 2OG-Fe(II) oxygenase superfamilies. Putative Fe^2+^ binding sites and conserved catalytic sites were also identifled. Phylogenetic analysis using a total of 15 *FLS* genes (Figure S2) showed that the *RrFLS1, PhFLS*, and *PpFLS* sequences clustered together with the *FLS* gene from *Arabidopsis* (Owens et al., 2008). This finding is consistent with the suggestion that each of the encoded gene products has a biochemically active role in flavonol synthesis.

Multiple alignments indicated that RrDFR1 and PhDFR showed high sequence identity with seven other DFR proteins. The alignment revealed that RrDFR1 and PhDFR have a highly conserved sequence including a putative NADPH binding domain (aa 10–30, VTGASGFIGSWLI/VMRLLEKGY) at the N terminus of DFR proteins, and also a motif predicted to be related to substrate specificity (Johnson et al., 1999, 2001) (Figure S3). The amino acid residue at position 134 (Figure S3) has been shown by Johnson et al. (2001) to directly influence substrate specificity. It is interesting to note, therefore, that *PhDFR* encodes an aspartic acid residue (D) at this position whereas, *RrDFR1* codes instead for asparagine (“N”). To further investigate the evolutionary relationship among DFRs, we built a rooted phylogenetic tree using the predicted amino acid sequences from 12 plant species (Figure S4).

### Overexpression of *FLS* increases flavonols and decreases anthocyanins in transgenic tobacco lines, and produces white flowers

**Figure 4:**
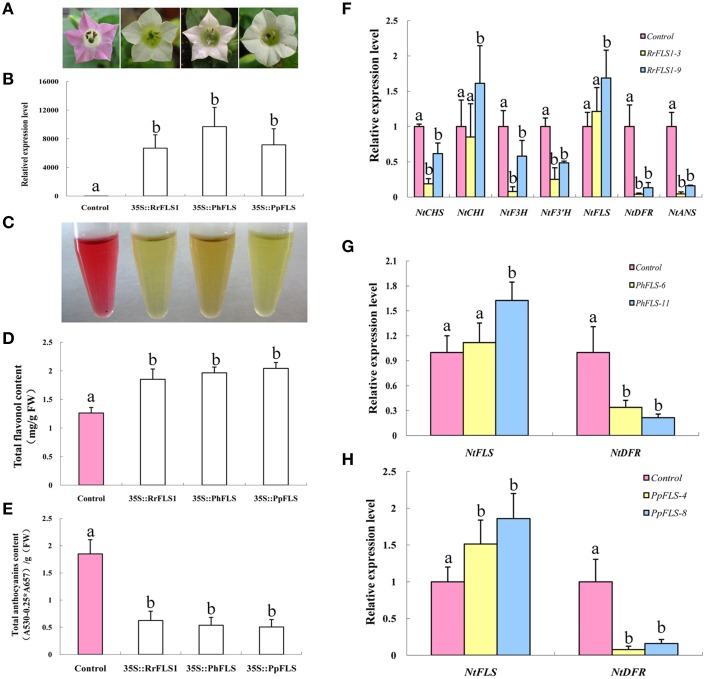
**Ectopic over-expression of *FLS* homologs in tobacco enhanced flavonol accumulation, depleted anthocyanin levels, and resulted in a white flower phenotype**. **(A)** Overexpression of *PhFLS, RrFLS1*, and *PpFLS* under the control of the cauliflower mosaic virus (CaMV) *35S* promoter in transgenic tobacco flowers resulted in a visible change in corolla pigmentation, as compared to the untransformed control. **(B)** Expression proflles of *PhFLS, RrFLS1*, and *PpFLS* genes in flowers of transgenic tobacco lines. **(C)** The extraction of pigment from flowers of transgenic tobacco lines. **(D)** Flavonol content in *RrFLS1, PhFLS, and PpFLS* transgenic and control flowers. **(E)** Anthocyanin content in *RrFLS1, PhFLS, and PpFLS* transgenic and control flowers. **(F)** Expression profiles of flavonoid-related structural biosynthetic genes in flowers of transgenic tobacco lines carrying *RrFLS1*gene. **(G)** Expression profiles of *NtFLS* and *NtDFR* genes in flowers of transgenic tobacco lines carrying *PhFLS* gene. **(H)** Expression profiles of *NtFLS* and *NtDFR* genes in flowers of transgenic tobacco lines carrying *PpFLS* gene. Different letters above the error bar indicate that values of the transgenic lines were significantly different from those of control at *P* < 0.05 using the Fisher's LSD test.

In order to test the *in vivo* function of the FLS sequences in flavonoid metabolism, the cloned sequences of *RrFLS1, PhFLS, and PpFLS* were ectopically expressed in stably-transformed tobacco plants. At least 10 transgenic tobacco lines were obtained for each construct (Table S1). In these experiments, we employed an early-flowering tobacco cultivar that normally shows pale pink flowers under the standard conditions supplied here within the growth chambers. However, plants harboring the *RrFLS1, PhFLS*, or *PpFLS* overexpression constructs displayed pure white in flower color and it was evident, even at the early flowering stages (Figure 4A, Figure S5). The *FLS*-transformed tobacco plants with this variant flower color contained significant levels of mRNA corresponding to the specific *RrFLS1, PhFLS*, or *PpFLS* constructs, whereas none was detected in control (non-transformed) plants (Figure 4B). It therefore seems reasonable to conclude that expression of the heterologous *FLS* gene was responsible for the change in flower pigmentation observed in these plants (Figure S6).

Flavonoids extraction results indicated that the tobacco flowers transformed with *FLSs* contained less anthocyanins (Figure 4C). HPLC analysis revealed that the flowers from each of these transgenic lines accumulated higher levels of flavonols and lower levels of anthocyanins than found in control flowers. In the petals of the *RrFLS1* lines, average flavonol content was measured to be approximately 68% higher than in control petals (Figure 4D). Similarly, in *PpFLS* lines the concentration of total flavonol was over 57% higher than in control plants (Figure 4D), and in *PhFLS* lines it was increased by approximately 31% (Figure 4D). Conversely, spectrophotometric analysis revealed that the anthocyanin content of the flowers of the *RrFLS1, PhFLS*, and *PpFLS* transgenic lines was significantly reduced (Figure 4E). Thus, in the *RrFLS1, PhFLS*, and *PpFLS* lines the anthocyanin content of the petals was decreased approximately 66, 74, and 59%, respectively, compared with that of the control flowers (Figure 4E). These results indicate that the *RrFLS1, PhFLS*, and *PpFLS* overexpression lead to an *in vivo* increase in flavonol accumulation and a significant decrease in anthocyanin content.

qRT-PCR method was used to examine the expression of other structural genes related to the flavonoid pathway in tobacco overexpressing *FLSs*. In the *RrFLS1* transgenic lines, the expression level of *NtCHI* was similar to that of control flowers whereas, the expression levels of *NtCHS, NtF3H*, and *NtF3*′*H* were decreased by 70–80% in the transgenic plants (Figure 4F). The strongest effect of the constitutive *RrFLS1* expression was measured in the abundance of *NtDFR* and *NtANS* transcripts, with *NtDFR* mRNA levels depressed by 99.6% in the transformed plants. By contrast, expression of *NtFLS* was increased by 20% (Figure 4F). In the *PhFLS* lines, *NtDFR* expression was decreased by 66.2%, and expression of *NtFLS* showed a10% increase compared to the control levels (Figure 4G). Similarly, in the *PpFLS* lines, the expression of *NtDFR* was markedly depressed (i.e., 99.3% decrease in transcript abundance), while expression of *NtFLS* was increased by 50% (Figure 4H). These results demonstrated that overexpression of the heterologous *FLS* genes in tobacco substantially inhibited expression of the *NtDFR* gene in the flowers and up-regulated expression of the endogenous *NtFLS* gene.

### Overexpression of *DFR* promotes the accumulation of anthocyanins in transgenic tobacco plants and produces red flowers

**Figure 5:**
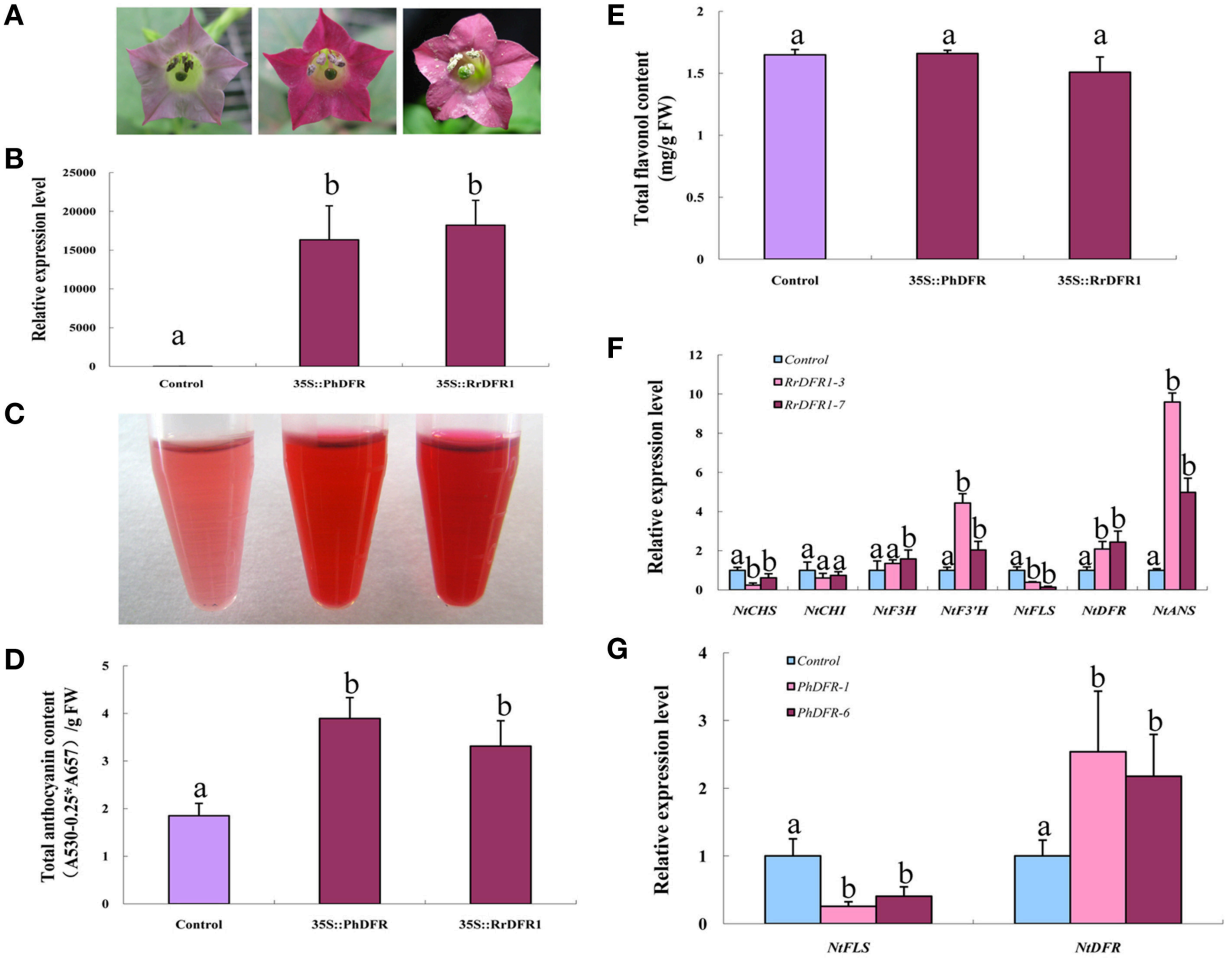
**Ectopic over-expression of *DFR* homologs in tobacco induced anthocyanin content, producing red flowers**. **(A)** Overexpression of *PhDFR* and *RrDFR1* under the control of the cauliflower mosaic virus (CaMV) *35S* promoter in transgenic tobacco flowers resulted in a visible increase in anthocyanin accumulation in the corolla, relative to control. **(B)** Expression proflles of *PhDFR* and *RrDFR1* genes in flowers of transgenic tobacco lines. **(C)** The extraction of pigment from flowers of transgenic tobacco lines carrying *PhDFR* or *RrDFR1* genes. **(D)** Anthocyanin levels in flowers of *PhDFR* and *RrDFR1* transgenic tobacco lines and in control flowers. **(E)** Flavonol levels in flowers of *PhDFR* and *RrDFR1* transgenic tobacco lines and in control flowers. **(F)** Expression proflles of flavonoid-related structural biosynthetic genes in flowers of transgenic tobacco lines carrying *RrDFR1*gene. **(G)** Expression profiles of *NtFLS* and *NtDFR* genes in flowers of transgenic tobacco lines carrying *PhDFR* gene. Different letters above the error bar indicate that values of the transgenic lines were significantly different from those of control at *P* < 0.05 using the Fisher's LSD test.

In order to test the function of the DFR proteins *in vivo*, we also over-expressed *RrDFR1* and *PhDFR* in tobacco plants. The flowers of the control (untransformed) tobacco plants typically developed pale pink petals. By comparison, the flowers of transgenic plants harboring the *RrDFR1* or *PhDFR* overexpression constructs appeared to be of a much darker pink color (Figure 5A). These *DFR* transgenic tobacco plants with variant flower color were shown to contain significant levels of *RrDFR1* or *PhDFR* mRNA, in contrast to control plants which contained none (Figure 5B). Thus, it seems reasonable to conclude that the increased *DFR* expression was responsible for the observed change in flower pigmentation. Metabolite extracts from the flowers confirmed the difference in visible flower pigmentation thus, the extracts of *DFR* transgenic plants were clearly deeper red in color than the lighter pink of the control flower extract (Figure 5C).

Spectrophotometric analysis demonstrated that the anthocyanin levels in the petals of *RrDFR1* and *PhDFR* transgenic lines were 2.1- and 1.8-fold, respectively, higher than those in the control flowers (Figure 5D). It should be noticed that no significant changes in flavonol content were detected between *DFR* transgenic plants and the control flowers (Figure 5E). Thus, it appears that the constitutive expression of heterologous *DFR* genes in tobacco differentially promoted the biosynthesis of anthocyanin.

qRT-PCR analysis was carried out to elucidate the effect of overexpression *DFRs* on flavonoids pathway in tobacco flowers. The relative expression levels of *NtCHS* and *NtFLS* were significantly down-regulated in the *RrDFR1*-transformed lines by 75.1 and 61.5%, respectively, and *NtDFR* and *NtANS* genes were upregulated 2- and 9.6-fold, respectively (Figure 5F). Similarly, transcript abundance of *NtFLS* in the *PhDFR* transformant was depressed by 74.2%, and expression of *NtDFR* was upregulated 2.5-fold, compared to the control (Figure 5G). Thus, these results demonstrated that the overexpression of the heterologous *DFR* genes in tobacco resulted in the endogenous *NtFLS* gene being significantly down-regulated, and concomitantly led to increased expression of the endogenous *NtDFR* gene.

### Relationship between the flavonoid biosynthesis gene expression and the anthocyanin and flavonol accumulation

**Figure 6:**
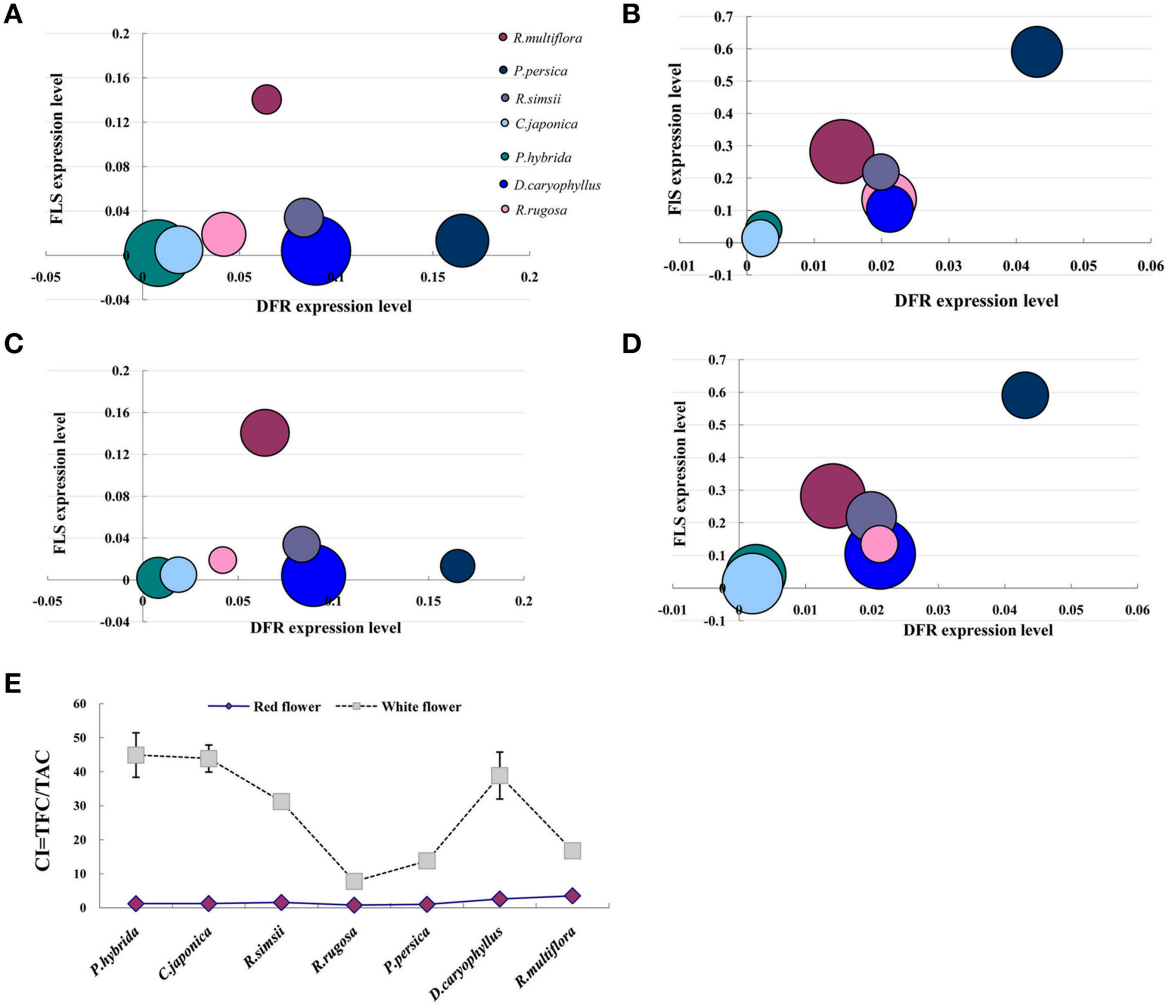
**Relationship between the flavonoid biosynthesis gene expression and the anthocyanin and flavonol accumulation**. **(A)** The amount of anthocyanin was used to compare with FLS and DFR expression levels in red flowers. **(B)** The amount of anthocyanin was used to compare with FLS and DFR expression levels in white flowers. **(C)** The amount of flavonol was used to compare with FLS and DFR expression levels in red flowers. **(D)** The amount of flavonol was used to compare with FLS and DFR expression levels in white flowers. **(E)** Difference in the copigmentation index (CI) between red and white flowers; TFC = total flavonoid content (μg/g FW), TFA = total anthocyanin content (μg/g FW). The size of bubbles represents the amount of flavonoid contents either anthocyanin or flavonol.

We investigated the relationship between the expression levels of *DFR* and *FLS* genes and the content of anthocyanin and flavonol, respectively, in petals of seven plant species (Figure 6). In the red flower, the higher expression level of *DFR* leaded to the accumulation of anthocyanin. However, the higher expression level of *FLS* in the white flower resulted in the accumulation of flavonol associated with the decreased expression of *DFR*. Furthermore, we calculated a value for the copigmentation index (CI) as the ratio of total flavonoid content (TFC) to total anthocyanin content (TAC). In each of the seven plant species studied, the white flowers demonstrated a significantly higher CI value than that for the respective red flowers (Figure 6E). Thus, our results demonstrate that flavonols form the predominant pigment type in white flower forms and are also present as co-pigments, alongside anothcyanins, in the red flower phenotype.

## Discussion

### FLS and DFR genes presents strong sequence conservation in plants

FLS and DFR genes isolated from rose, petunia and peach encode functional proteins. In this study, the *RrFLS1, PpFLS*, and *PhFLS* genes were found to share striking sequence identity with *FLS* sequences previously isolated from other plant species, with the middle portion of the encoded polypeptide sequence indicating particularly strong sequence conservation. The isolated *FLS* genes contained a highly conserved N-terminal domain with motifs typical of the 2OG-Fe(II) dioxygenase superfamily and 2OG-Fe(II) oxygenase superfamily (Figure S1). The presence of these highly conserved motifs suggested that RrFLS1, PpFLS and PhFLS cloned in our study shared striking sequence similarity with other FLSs and may have a biological function same as or similar to them in flavonol sythesis, and this conclusion was strongly supported by the measurement of increased flavonol levels in transgenic tobacco plants expressing these heterologous constructs. The phylogenetic tree revealed relationship between RrFLS1, PpFLS, PhFLS, and FLSs from other plants, in which RrFLS1, PpFLS, and PhFLS was closely related to AtFLS1 from *Arabidopsis thaliana*. AtFLS1 has been shown to induce the accumulation of the flavonol, implying that *RrFLS1, PpFLS, and PhFLS* might function in the same way as FLSs of *Arabidopsis*. *RrFLS1, PpFLS, and PhFLS* sharing a high degree of sequence similarity and identity with other plant FLSs determined the conserced biological functions in varied plants.

Amongst the various flavonoid metabolites of higher plants, the anthocyanin biosynthesis pathway has been shown to be the most closely associated with the formation of flower color (Quattrocchio et al., 2006; Yamagishi et al., 2010). A key step in anthocyanin synthesis is mediated by dihydroflavonol-4-reductase, and we found that the *RrDFR1* and *PhDFR* genes shared a high degree of sequence similarity and identity with other known plant *DFR* genes. In each case, the N terminus of the encoded DFR protein contained a putative NADPH binding domain (aa 10–30, VTGASGFIGSWLI/VMRLLEKGY) (Peters and Constabel, 2002). The presence of such highly conserved motifs indicated that *RrDFR1* and *PhDFR* potentially encoded functional enzymes for the conversion of dihydroflavonols into the precursors of anthocyanins (Shimada et al., 2005; Lo-Piero et al., 2006). The increased anthocyanin content detected in the transgenic tobacco lines expressing these heterologous genes strongly supported this conclusion.

### Metabolites are related to the formation of flower color

Flavonoids are widely distributed amongst the plant species and, in particular, anthocyanins and flavonols are the most important pigments for flower color determination, leading to a range of colors including white, yellow, red, and purple (Tanaka et al., 2008; Morita et al., 2014). In this study, we demonstrated that, amongst seven diverse plant species, the anthocyanin content of red flowers was consistently much higher than that of the respective white flowers. Conversely, flavonols comprised the major pigment in white flowers, with anthocyanin levels below the limits of our detection. Interestingly, the total content of flavonols was found not to differ significantly between the red and white flower phenotypes. It has been suggested that the flavonol branch of the pathway has remained intact for millions of years, and is important in enabling the plant to respond to a multitude of stresses (Pollastri and Tattini, 2011). In red flowers, we found flavonols to be present as one of the co-pigments, but analyses of *FLS* mutants have indicated that such a contribution to petal color is limited to magenta-through-to-blue phenotypes (Holton et al., 1993; Pollak et al., 1993; Takahashi et al., 2007). Transgenic tobacco lines expressing an introduced *FLS* gene construct demonstrated that over-expression of *FLS* could transform petals from having a pink pigmentation to appearing completely white. This color change was concomitant with the increased accumulation of flavonols and a reduction in anthocyanin content. In contrast, transgenic tobacco lines expressing an introduced *DFR* gene accumulated increased levels of anthocyanins, but there was little or no change in flavonol accumulation as a result of this *DFR* over-expression. These findings in genetically-transformed tobacco plants with regard to flavonoid levels and flower color were consistent with the *in vivo FLS* and *DFR* gene functions elucidated within the seven original plant species. Interestingly, although the expression of *NtFLS* was repressed in the *DFR* over-expressing transgenic plants, the concentration of flavonols did not change significantly, and we suggest that this may indicate that FLS enzymic activity was originally in excess of that required to maintain flavonol levels. Based on these variations in metabolite levels, we conclude that flavonols contribute significantly to achieving the white flower pigmentation, and that the accumulation of anthocyanins is the key factor in determining the red flower phenotype with flavonols present only as a co-pigment. However, it remains to be established how the precise ratio of flavonol and anthocyanin metabolites affects the formation of flower color in plants.

### The competition between FLS and DFR genes determines flower color

To examine the transcriptional control of flavonol and anthocyanin accumulation in the red and white flower phenotypes, real-time PCR was used to follow the expression of six genes of the flavonoid biosynthesis pathway within seven plant species. Of these six genes, only *FLS* (encoding an important enzyme of the flavonoid pathway that catalyzes the formation of flavonols) and *DFR* (encoding a critical enzyme in the regulation of anthocyanin biosynthesis) exhibited distinct differential expression patterns between the white and red flower colors. Thus, *FLS* expression levels were substantially higher in the white flowers whereas, *DFR* expression levels were significantly higher in the red flowers. Furthermore, it was found in white flowers that *FLS* transcript levels exceeded those of *DFR* whereas, in red flowers *DFR* transcript abundance exceeded *FLS* levels. These different expression patterns of *DFR* and *FLS* genes contributed toward establishing the competition between DFR and FLS enzymes for common substrates of the anthocyanin and flavonol branches of the flavonoid biosynthetic pathway.

In this study, we showed that the ectopic expression of a heterologous *FLS* gene led to the significant down-regulation of endogenous *NtDFR* expression in genetically transformed tobacco lines, and promoted *NtFLS* expression albeit weakly. Conversely, the overexpression of introduced *DFR* genes suppressed the expression of *NtFLS* and promoted the expression of *NtDFR*. It is very interesting that over-expression of *FLS* or *DFR* could repress each other. On the one hand, the expression of *NtFLS* and *NtDFR* could be regulated by feedback mechanism (Loake et al., 1991; Yin et al., 2012; Liu et al., 2013; Liao et al., 2015). In other words, the accumulation of flavonol in tobacco inhibited the expression *NtDFR*. In turn, high content of anthocyanins suppressed the *NtFLS* expression. For example, overexpression of *MdLAR1* in tobacco resulted in the catechin accumulation and the decreased expression of *NtANS* (Liao et al., 2015). On the other hand, FLS and DFR have the same substrate-dihydroflavonol, which is the most important branch point in the flavonoid pathway. Once the FLS was enhanced, the substrate was taken by it, DFR has less chance to catalyze the substrate, the metabolic flux conduct to the flavonol. Over-expression DFR also worked in the same way. Further, it is well known that R2R3-MYB transcription factors (TFs) have played an important role in the biosynthesis of anthocyanins and flavonols. It has been previously reported that overexpression of *AtMYB12* in tobacco leaded to the accumulation of flavonol and the enhanced the expression of *FLS* (Luo et al., 2008). In apple, MYB10 was involved in the accumulation of anthocyanins by induced the expression of DFR (Jiang et al., 2014). Thus, the changes of expression level of MYB could regulate the flavonoids synthesis via modulating the expression of genes related to the flavonol and anthocyanin pathway.

### Differential expression of FLS and DFR correlates with flavonol and anthocyanin concentrations

The visible accumulation of anthocyanins in red flowers typically reflects the activity of biosynthetic enzymes functioning in the anthocyanin branch of the flavonoid pathway. In red flowers, expression of the DFR gene is higher than that of FLS, which is consistent with the accumulation of anthocyanins. In white flowers, our results showed that mRNA levels encoding the FLS enzyme were higher than transcript levels encoding DFR, and this expression pattern correlated with the dominant accumulation of flavonols. Interestingly, expression of FLS in white flowers was found to be higher than in red flowers, but this pattern was not reflected in the total levels of flavonol which did not differ between the two color forms. Expression of DFR, on the other hand, was significantly higher in red flowers compared to white flowers, and this pattern was reflected in the respective levels of anthocyanin. Complementary results were obtained in the FLS and DFR transgenic experiments. Thus, overexpression of FLS in tobacco inhibited expression of *NtDFR* and resulted in the predominant accumulation of flavonols giving the flowers a pure white appearance. Overexpression of *DFR* in transgenic tobacco plants led to down-regulated expression of *NtFLS*, so leading to the accumulation of anthocyanins as the dominant flavonoid and resulting in flowers with a deep red coloration. It is should be noticed that over-expression of *FLS* or *DFR* leads to the down-regulation of the genes that are involved in the early step of flavonoid biosynthesis. This phenomenon could be explained that feedback mechanism existing in the flavonoid pathway had an effect on the expression of gene associated with early step of flavonoid biosynthesis (Loake et al., 1991; Yin et al., 2012; Liao et al., 2015). The results obtained in this study provide further supporting evidence for the inverse correlation between flavonol and anthocyanin production, and the importance of the competing expression patterns of *FLS* and *DFR* in determining flower color phenotype.

**Figure 7:**
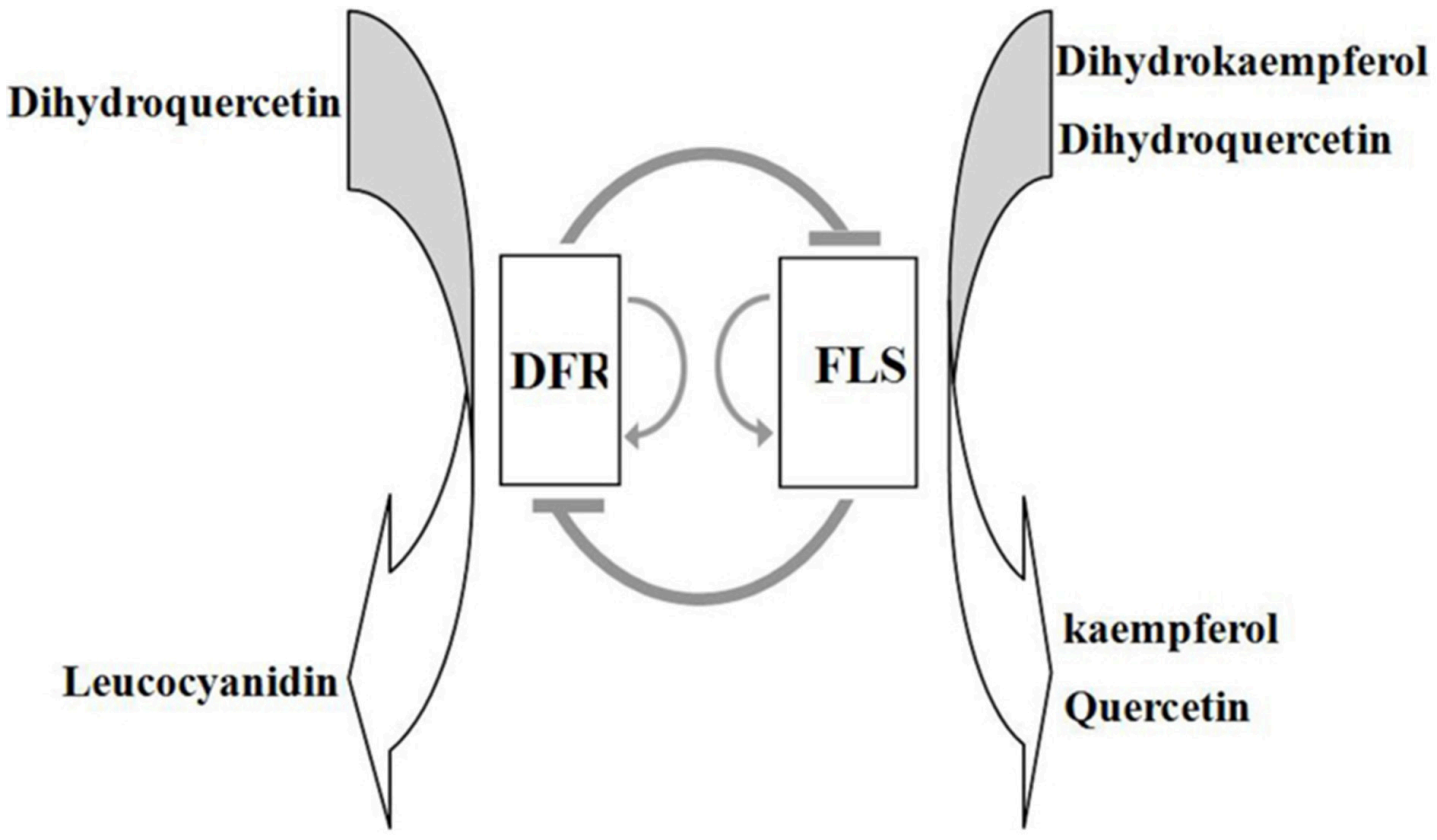
**Proposed model for the interaction of *DFR* and *FLS* genes and their products in the control of biosynthesis of flavonols and anthocyanins**. In this model, expression of *DFR* and *FLS* genes mutually suppresses transcription of the other gene, whilst promoting self-expression; this scenario was supported by the findings in *DFR* and *FLS* overexpressing transgenic lines.

We report the findings of an investigation into the relationship between flavonol and anthocyanin accumulation, and the associated expression patterns of flavonoid biosynthetic genes in the red and white flower phenotypes of seven plant species. The combined results of various experimental scenarios clearly showed that the competition between *FLS* and *DFR* genes ultimately determines the formation of flower color. In transgenic experiments, overexpression of heterologous *FLS* genes in tobacco inhibited the expression of *NtDFR*, thereby leading to the accumulation of flavonols as the major flower pigments which conferred the phenotype of pure white petals. Conversely, overexpression of *DFR* in transgenic tobacco plants caused down-regulated expression of *NtFLS*, leading to the accumulation of anthocyanins and a deep red flower phenotype. Thus, we conclude that the enzymic products of the *FLS* and *DFR* genes compete for common substrates in order to direct the biosynthesis of flavonols and anthocyanins, respectively, and the outcome of this competition is ultimately responsible for the formation of the red flower vs. white flower phenotypes. We present a schematic model to describe this competitive interaction of flavonol and anthocyanin biosynthetic pathways in flowers (Figure 7).

## Author contributions

MB, PL, and GN conceived the study and participated in its design. GN and ZW provided the materials for the study. PL and YS performed the Real-time PCR. PL, HJ, JZ, and PL performed the HPLC analysis. PL and GN performed the construction of expression vectors and genetic transformation. PL and GN wrote the manuscript. All authors read and approved the final manuscript.

### Conflict of interest statement

The authors declare that the research was conducted in the absence of any commercial or financial relationships that could be construed as a potential conflict of interest.

## Supplementary material

Figure S1 **Alignment of a highly conserved region of FLS amino acid sequences**. Multiple alignments of the predicted protein sequence of FLSs from eight plants, including *Rosa rugosa* (RrFLS1, KM099095), *Rosa multiflora* (RmFLS, KP090455), *Prunus persica* (PpFLS, KP050782), *Petunia hybrida* (PhFLS, Q07512.1), *Nicotiana tabacum* (NtFLS1, ABE28017.1), *Arabidopsis thaliana* (AtFLS1, NM_001203337.1), *Dianthus caryophyllus* (DcFLS, KM203112), *Camellia nitidissima* (CjFLS, JF343560.1). Red line indicated highly conserved N-terminal region of proteins with 2-oxoglutarate/Fe(II)-dependent dioxygenase activity; green, 2OG-Fe(II) oxygenase superfamily domain. Red asterisks indicate 2-ODD superfamily conserved residues. The Fe^2+^ binding sites were indicated by blue arrowheads; green arrowheads indicate conserved catalytic sites.

Figure S2 **A phylogenetic tree constructed based on the amino acid sequences of 15 FLSs, including RrFLS1 (KM099095), PpFLS (KP050782), RmFLS (KP090455), GmFLS (NP_001237419.1), CnFLS (ADZ28516.1), AmFLS (ABB53382.1), PhFLS (Q07512.1), NtFLS1 (ABE28017.1), AtFLS1 (NM_001203337.1), CsFLS (ABM88786.1), VvFLS (BAE75810.1), GbFLS (ACY00393.1), DcFLS (KM203112), MtFLS (XP_003613111.1), CaFLS (XP_004489776.1)**. Note: *RrFLS1, PhFLS*, and *PpFLS* sequences clustered together with the *FLS* gene from *Arabidopsis*. The numbers beside the branches represent bootstrap values based on 1000 replications, and the relative amount of change along branches is indicated by the scale bar. The analysis was performed with MEGA 4 based on the neighbor-joining method.

Figure S3 **Alignment of a highly conserved region of DFR amino acid sequences**. Multiple alignments of the predicted protein sequence of DFRs from nine plants, including Rosa rugosa (RrDFR1, KM203111), Petunia hybrida (PhDFR, AF233639), Rosa mulriflora (RmDFR, KP137549), *Arabidopsis thaliana* (AtDFR, NM_123645.3), Camellia sinensis (CsDFR, AB018686.1), Prunus persica (PpDFR, HM543571.1), Rhodendron simsii (RsDFR, CAC88859.1), Dianthus caryophyllus (DcDFR, AF291097.1), Nicotiana tabacum (NtDFR1, EF421429.1). *RrDFR1* and *PhDFR* contain a highly conserved sequence including a putative NADPH binding domain (aa 10–30, VTGASGFIGSWLI/VMRLLEKGY) at the N terminus, and also a motif predicted to be related to substrate specificity (Johnson et al., 1999, 2001).

Figure S4 **Phylogenic tree analysis of the predicted amino acid sequences of DFR polypeptides from 12 plant species, including RrDFR1 (KM203111), RmDFR (KP137549), PpDFR (HM543571.1), VvDFR (CAA53578.1), CsDFR (AB018686.1), DcDFR (AF291097.1), AtDFR (NM_123645.3), CsDFR (AB018686.1), AmDFR (CAA33543.1), GtDFR (BAA12736.1), PhDFR (AF233639), NtDFR1(EF421429.1)**. The numbers beside the branches represent bootstrap values based on 1000 replications, and the relative amount of change along branches is indicated by the scale bar. The analysis was performed with MEGA 4 based on the neighbor-joining method.

Figure S5 **Structure of the gene construct used to express heterologous *FLS* and *DFR* genes in transgenic tobacco lines**.

Figure S6 **Phenotypic comparisons of *DFR* and *FLS* transgenic tobacco plants**.

Table S1 **The quantitative traits of transgenic tobacco plants expressing heterologous *DFR* or *FLS* genes**.

Table S2 **Primer sequences used for the isolation of complete *RrDFR1, PhDFR, RrFLS1, PpFLS*, and *PhFLS* coding sequences**.

Table S3 **Primer sequences for qRT-PCR analyses**.
