# Supplementary material for: Disequilibrium of Flavonol Synthase and Dihydroflavonol-4-Reductase Expression Associated Tightly to White vs. Red Color Flower Formation in Plants
Source: Front Plant Sci. 2016 Jan 13;6:1257. doi: 10.3389/fpls.2015.01257 (PMC4710699; doi:10.3389/fpls.2015.01257)
Supplement: Figure S1 — Alignment of a highly conserved region of FLS amino acid sequences. Multiple alignments of the predicted protein sequence of FLSs from eight plants, including Rosa rugosa (RrFLS1, KM099095), Rosa multiflora (RmFLS, KP090455), Prunus persica (PpFLS, KP050782), Petunia hybrida (PhFLS, Q07512.1), Nicotiana tabacum (NtFLS1, ABE28017.1), Arabidopsis thaliana (AtFLS1, NM_001203337.1), Dianthus caryophyllus (DcFLS, KM203112), Camellia nitidissima (CjFLS, JF343560.1). Red line indicated highly conserved N-terminal region of proteins with 2-oxoglutarate/Fe(II)-dependent dioxygenase activity; green, 2OG-Fe(II) oxygenase superfamily domain. Red asterisks indicate 2-ODD superfamily conserved residues. The Fe2+ binding sites were indicated by blue arrowheads; green arrowheads indicate conserved catalytic sites. [file Image1.PDF]

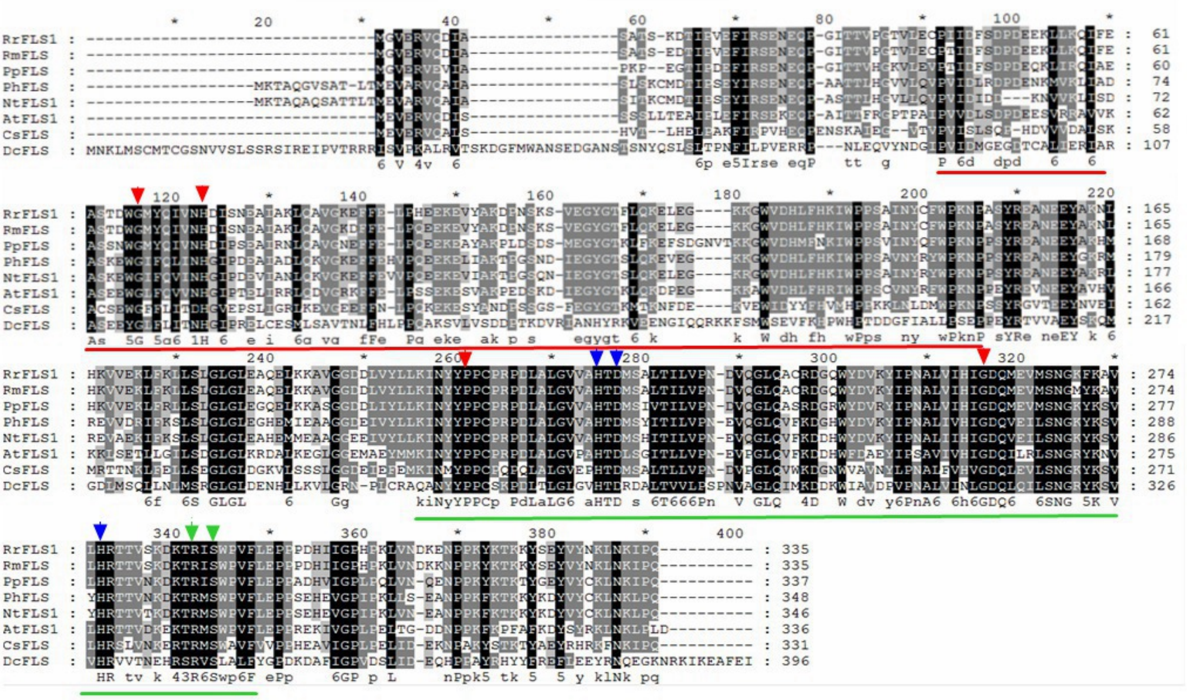
RrFLS1
RmFLS
PpFLS
PhFLS
NtFLS1
AtFLS1
CsFLS
DcFLS
