# Supplementary figures and images for: Disequilibrium of Flavonol Synthase and Dihydroflavonol-4-Reductase Expression Associated Tightly to White vs. Red Color Flower Formation in Plants
Source: Front Plant Sci. 2016 Jan 13;6:1257. doi: 10.3389/fpls.2015.01257 (PMC4710699; doi:10.3389/fpls.2015.01257)

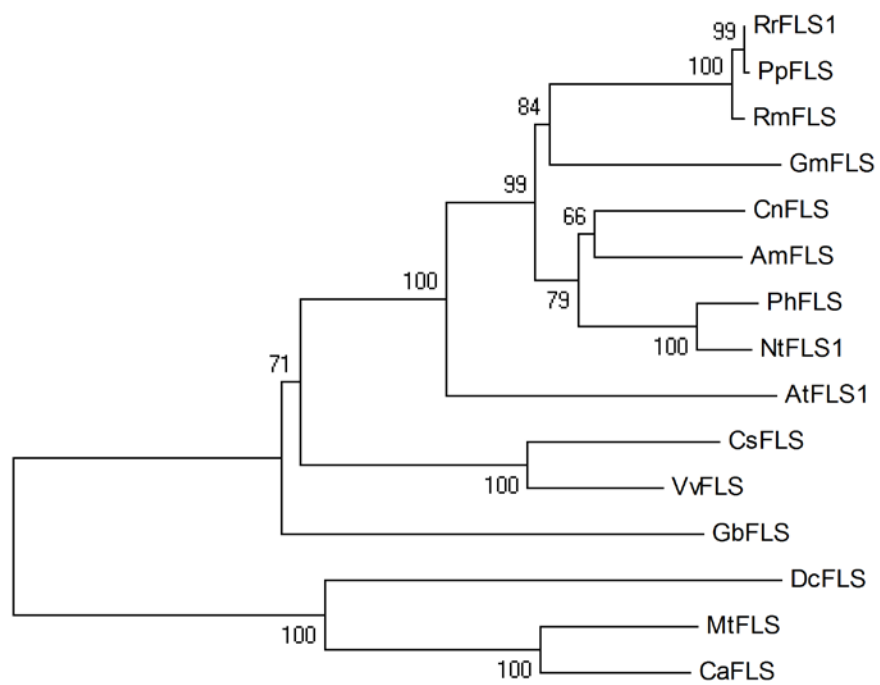

0.1

Supplement: Figure S2 — A phylogenetic tree constructed based on the amino acid sequences of 15 FLSs, including RrFLS1 (KM099095), PpFLS (KP050782), RmFLS (KP090455), GmFLS (NP_001237419.1), CnFLS (ADZ28516.1), AmFLS (ABB53382.1), PhFLS (Q07512.1), NtFLS1 (ABE28017.1), AtFLS1 (NM_001203337.1), CsFLS (ABM88786.1), VvFLS (BAE75810.1), GbFLS (ACY00393.1), DcFLS (KM203112), MtFLS (XP_003613111.1), CaFLS (XP_004489776.1). Note: RrFLS1, PhFLS, and PpFLS sequences clustered together with the FLS gene from Arabidopsis. The numbers beside the branches represent bootstrap values based on 1000 replications, and the relative amount of change along branches is indicated by the scale bar. The analysis was performed with MEGA 4 based on the neighbor-joining method. [file Image2.PDF]

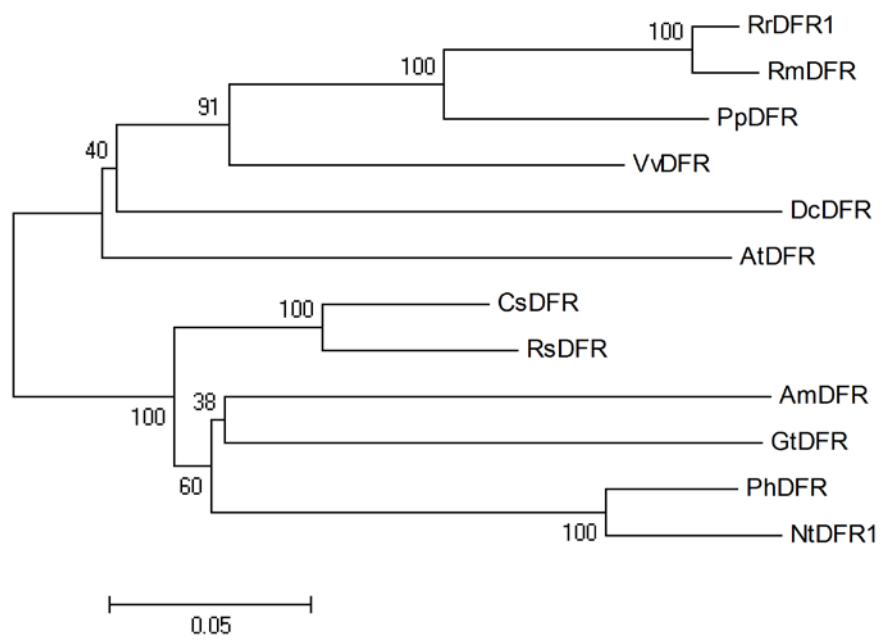

Supplement: Figure S4 — Phylogenic tree analysis of the predicted amino acid sequences of DFR polypeptides from 12 plant species, including RrDFR1 (KM203111), RmDFR (KP137549), PpDFR (HM543571.1), VvDFR (CAA53578.1), CsDFR (AB018686.1), DcDFR (AF291097.1), AtDFR (NM_123645.3), CsDFR (AB018686.1), AmDFR (CAA33543.1), GtDFR (BAA12736.1), PhDFR (AF233639), NtDFR1(EF421429.1). The numbers beside the branches represent bootstrap values based on 1000 replications, and the relative amount of change along branches is indicated by the scale bar. The analysis was performed with MEGA 4 based on the neighbor-joining method. [file Image4.PDF]

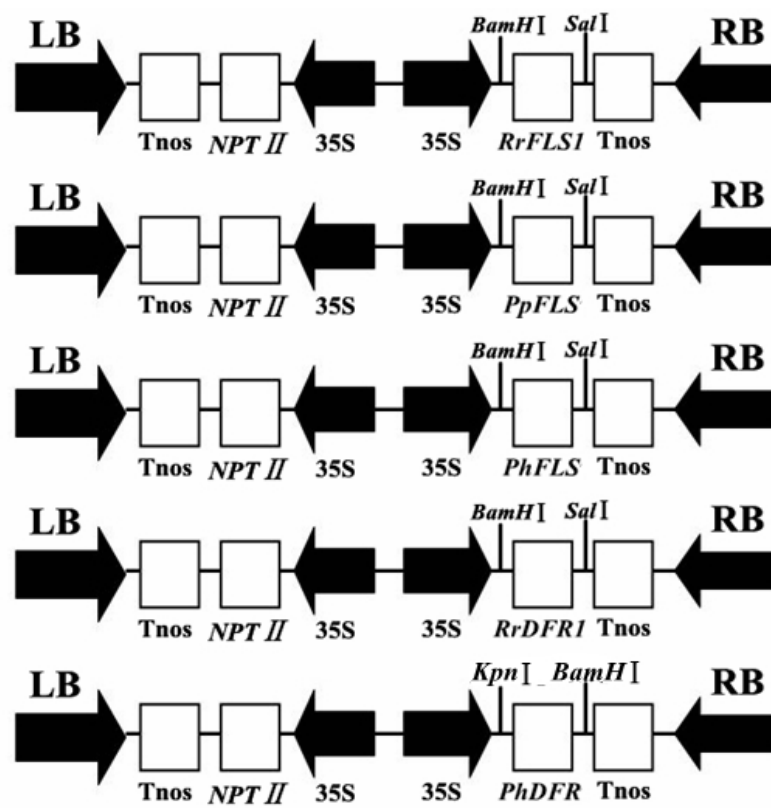

Supplement: Figure S5 — Structure of the gene construct used to express heterologous FLS and DFR genes in transgenic tobacco lines. [file Image5.PDF]

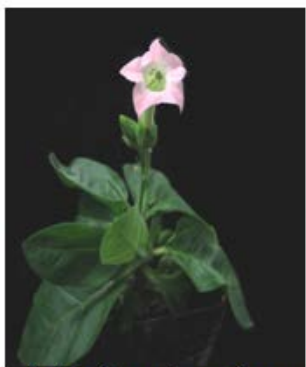

Early-flowering tobacco

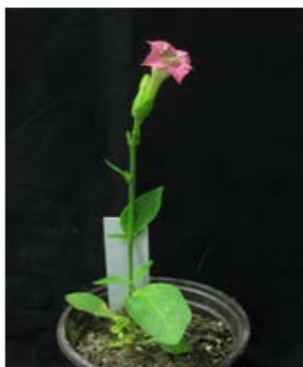

35S::RrDFR1

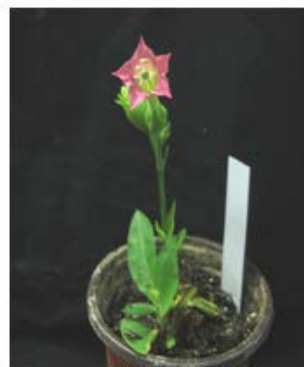

35S::PhDFR

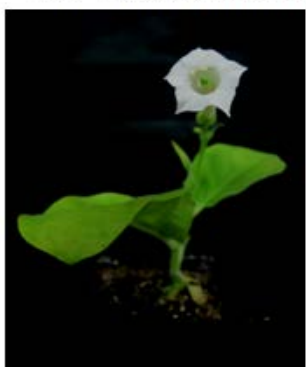

35S::RrFLS1

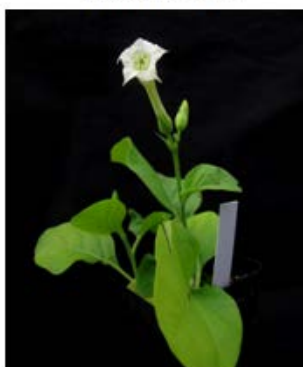

35S::PhFLS

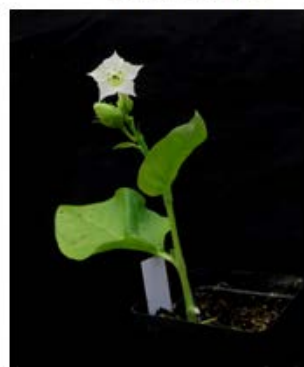

35S::PpFLS

Supplement: Figure S6 — Phenotypic comparisons of DFR and FLS transgenic tobacco plants. [file Image6.PDF]
