# Supplementary material for: Disequilibrium of Flavonol Synthase and Dihydroflavonol-4-Reductase Expression Associated Tightly to White vs. Red Color Flower Formation in Plants
Source: Front Plant Sci. 2016 Jan 13;6:1257. doi: 10.3389/fpls.2015.01257 (PMC4710699; doi:10.3389/fpls.2015.01257)
Supplement: Figure S3 — Alignment of a highly conserved region of DFR amino acid sequences. Multiple alignments of the predicted protein sequence of DFRs from nine plants, including Rosa rugosa (RrDFR1, KM203111), Petunia hybrida (PhDFR, AF233639), Rosa mulriflora (RmDFR, KP137549), Arabidopsis thaliana (AtDFR, NM_123645.3), Camellia sinensis (CsDFR, AB018686.1), Prunus persica (PpDFR, HM543571.1), Rhodendron simsii (RsDFR, CAC88859.1), Dianthus caryophyllus (DcDFR, AF291097.1), Nicotiana tabacum (NtDFR1, EF421429.1). RrDFR1 and PhDFR contain a highly conserved sequence including a putative NADPH binding domain (aa 10–30, VTGASGFIGSWLI/VMRLLEKGY) at the N terminus, and also a motif predicted to be related to substrate specificity (Johnson et al., 1999, 2001). [file Image3.PDF]

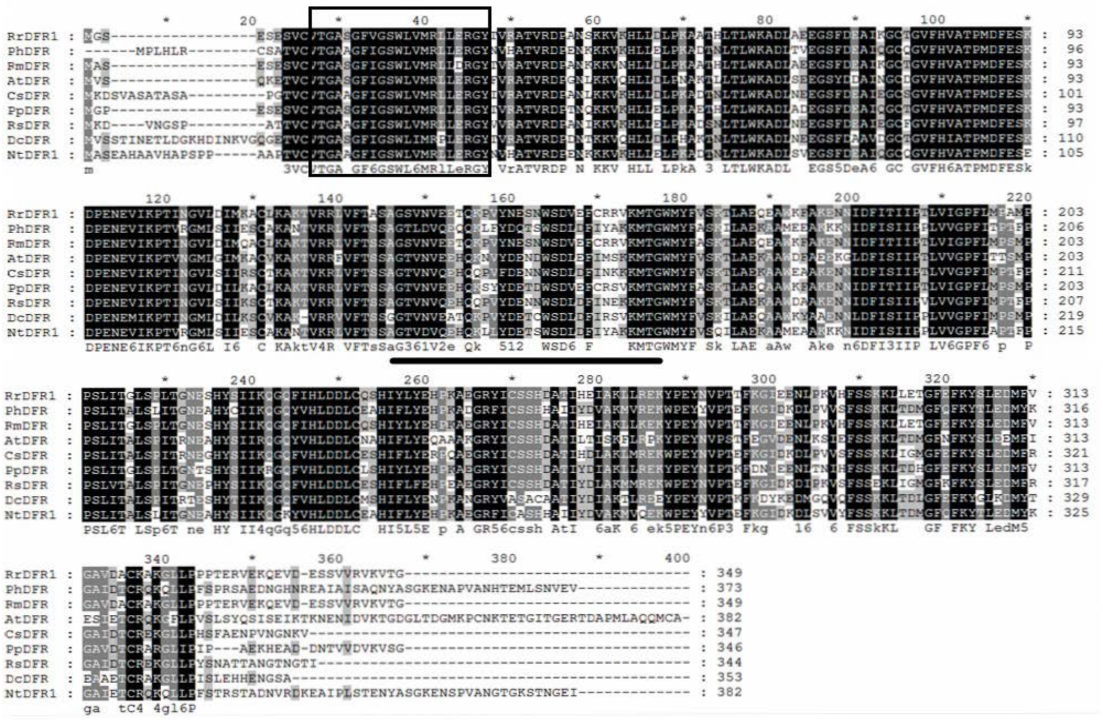

RrDFR1
PhDFR
RmDFR
AtDFR
CsDFR
PpDFR
RsDFR
DcDFR
NtDFR1
