# Supplementary material for: Disequilibrium of Flavonol Synthase and Dihydroflavonol-4-Reductase Expression Associated Tightly to White vs. Red Color Flower Formation in Plants
Source: Front Plant Sci. 2016 Jan 13;6:1257. doi: 10.3389/fpls.2015.01257 (PMC4710699; doi:10.3389/fpls.2015.01257)
Supplement: Table S1 — The quantitative traits of transgenic tobacco plants expressing heterologous DFR or FLS genes. [file Table1.DOC]

**Table S1.** The quantitative traits of transgenic tobacco plants expressing heterologous *DFR* or *FLS* genes.

| Type | Total | Strong | Medium | Weak | Positive rate (%) |
| --- | --- | --- | --- | --- | --- |
| 35S::RrDFR1 | 35 | 12 | 8 | 7 | 77.1 |
| 35S::PhDFR | 32 | 11 | 9 | 6 | 81.3 |
| 35S::RrFLS1 | 36 | 10 | 13 | 9 | 88.9 |
| 35S::PhFLS | 33 | 12 | 9 | 6 | 81.8 |
| 35S::PpFLS | 35 | 9 | 12 | 10 | 88.6 |
