# Supplementary material for: Disequilibrium of Flavonol Synthase and Dihydroflavonol-4-Reductase Expression Associated Tightly to White vs. Red Color Flower Formation in Plants
Source: Front Plant Sci. 2016 Jan 13;6:1257. doi: 10.3389/fpls.2015.01257 (PMC4710699; doi:10.3389/fpls.2015.01257)
Supplement: Table S2 — Primer sequences used for the isolation of complete RrDFR1, PhDFR, RrFLS1, PpFLS, and PhFLS coding sequences. [file Table2.DOC]

**Table S2.** Primer sequences used for the isolation of complete *RrDFR1*, *PhDFR*, *RrFLS1*, *PpFLS* and *PhFLS* coding sequences.

| Genus | Gene | Primer sequence |
| --- | --- | --- |
| *Rosa rugosa* | RrDFR1qF | **GGATCC**ATGGGATCGGAATCCGAG |
|  | RrDFR1qR | **GTCGAC**TTAGCCTGTGACTTTGACACG |
|  | RrFLS1qF | **GGATCC**ATGGGGGTAGAGAGAGTTCAAG |
|  | RrFLS1qR | **GTCGAC**TTACTGGGGGATCTTGTTGA |
| *Petunia hybrida* | PhDFRqF | **GGTACC**ATGCCCCTTCACCTCCGG |
|  | PhDFRqR | **GGATCC**CTAGACTTCAACATTGCTTAACATTTC |
|  | PhFLSqF | **GGATCC**AGAAAATGAAAACAGCTCAAGGT |
|  | PhFLSqR | **GTCGAC**TCGCTCCATACATAGAGGTGC |
| *Prunus persica* | PpFLSqF | **GGATCC**ATGGGGGTAGAGAGAGTCGAG |
|  | PpFLSqR | **GTCGAC**TTATTGGGGAATTTTGTTGAGC |
