# Supplementary material for: Disequilibrium of Flavonol Synthase and Dihydroflavonol-4-Reductase Expression Associated Tightly to White vs. Red Color Flower Formation in Plants
Source: Front Plant Sci. 2016 Jan 13;6:1257. doi: 10.3389/fpls.2015.01257 (PMC4710699; doi:10.3389/fpls.2015.01257)
Supplement: Table S3 — Primer sequences for qRT-PCR analyses. [file Table3.DOC]

**Table S3.** Primer sequences for qRT-PCR analyses.

| Genus | Gene | Primer sequence |
| --- | --- | --- |
| *Rosa rugosa* | RrCHSF | GCCTGAAACCCGAAAAGTTAGAA |
|  | RrCHSR | CAAAACCAAATAGGACACCCCAC |
|  | RrCHIF | ATGCCATCTCTCCAAGAATCAAAT |
|  | RrCHIR | AGAACACATCAAAAAGTGCCGAA |
|  | RrF3HF | AGGTTGTCCATAGCCACATTCCA |
|  | RrF3HR | AGGTCCTTGCTCATCTTCTTCTTGT |
|  | RrF3’HF | ATCAAGCACGGTGGAATGGG |
|  | RrF3’HR | GGATGGGTGGAGTCGGAACG |
|  | RrDFR1F | CAAGGGCATTGAGGAGAACTTGC |
|  | RrDFR1R | CCTGTGACTTTGACACGGACGA |
|  | RrFLS1F | TGGAGGGATACGGAACATTTTTA |
|  | RrFLS1R | CACCACCTTGTGTAGATTCTTTGC |
|  | RrGAPDHF | TGAAGGGTGGTGCCAAGAA |
|  | RrGAPDHR | AAGGGGAGCAAGACAGTTGG |
| *Petunia hybrida* | PhCHSF | CAAGCACTTTTTGGTGATGGGG |
|  | PhCHSR | TTCCTCAAGGCTCTTCTCAATGTTT |
|  | PhCHIF | CCGAAAAATGGAAAGGCAAAA |
|  | PhCHIR | CTTCTCAATGGCACGACCCTC |
|  | PhF3HF | AGTGGGTAACTGCTAAGTGTGTGC |
|  | PhF3HR | CAGTGACAGTCTCAGGTAGGGGC |
|  | PhF3’HF | ATGTCCGCCAGGATGAAGTGAA |
|  | PhF3’HR | TGACTTGAACTCCGCCGCTTGT |
|  | PhDFRF | CCTGCCAGTGGTGTCTTTTTCA |
|  | PhDFRR | TTCTCGGTTATGTCCATTGTCTTCA |
|  | PhFLSF | AATGAGCAACCAGCAGCCAC |
|  | PhFLSR | TCTTTCCCTACTTTCTGTAAATCCG |
|  | PhactinF | GTTGGACTCTGGTGATGGTGTG |
|  | PhactinR | CCGTTCAGCAGTGGTGGTG |
| *Prunus persica* | PpCHSF | AGGGTGCTCGTGTTCTCGTTGT |
|  | PpCHSR | CTGTCGGGAAGGATGGTTTGG |
|  | PpCHIF | GACAACGATACTGCCACTAACCG |
|  | PpCHIR | AAGACCTCAAGGAACTTCTCAATG |
|  | PpF3HF | CTGTCCATAGCCACATTCCAAAAC |
|  | PpF3HR | GCCCTCCTTCTCCGAGTCCTG |
|  | PpF3’HF | AAAGAGGATGCTGACGGTGAGG |
|  | PpF3’HR | GGTGGCGAAGGAGTTCTGCTAT |
|  | PpDFRF | ATCAAGCCAACAATAAATGGGGTG |
|  | PpDFRR | TCGCTCCAGTCGGTTTCGTC |
|  | PpFLSF | TGTCCATCGTCACCATTCTCGT |
|  | PpFLSR | TCCATTGCTCATTACCTCCATTTG |
|  | PpactinF | TCCGAGAAGATGACCCAAATAATG |
|  | PpactinR | CACCAGAATCCAGCACAATACCA |
| *Rhodendron simsii* | RsCHSF | CGGATTACTAACAGCGAGCACAAG |
|  | RsCHSR | ACACACACTGGGATTTTCCTTCA |
|  | RsCHIF | CTTTAACGGGCAAGGCATACTCAG |
|  | RsCHIR | CGTTAGCGACCCAAGAGGTGATT |
|  | RsF3HF | TGCCCACAACCCGACCTC |
|  | RsF3HR | CTTGAACCTCCCGTTGCTTAGAT |
|  | RsF3’HF | CACGACCGTAAATCTCGGACAAC |
|  | RsF3’HR | GGTACGAAGTCGCCGATATTGA |
|  | RsDFRF | GAGGCTGGTGTTCACATCTTCTG |
|  | RsDFRR | GCTGCTTTCTCTGCTAGTGTTTTTG |
|  | RsFLSF | ATGTCCGCTCTCACCCTGCT |
|  | RsFLSR | TCCTTGTTCGTTCCTTGTCCAC |
|  | RsactinF | TGAGACTTTCAATGTTCCAGCAATG |
|  | RsactinR | ACGACCAGCAAGATCAAGACGG |
| *Nicotiana tabacum* | NtCHSF | AAGCAAGAGAAACTAAAGGCTACAAG |
|  | NtCHSR | AAATCCAAAAAGCACACCCCAT |
|  | NtCHIF | CGGGTGCCTCCATTCTTTTTACT |
|  | NtCHIR | CCTGACACTCTTTCGGCGATACTAC |
|  | NtF3HF | CCAGACAAACCAGATGGATGGATAG |
|  | NtF3HR | CAAGGGTAAGGTCGGGCTGTG |
|  | NtF3’HF | TGGCTATTTCATTCCAAAAGGCTCA |
|  | NtF3’HR | CTTCAAAGTCATTTCCTCGCACATC |
|  | NtDFRF | GCAGTTGCTTCCCTTTTCTACC |
|  | NtDFRR | TTCCCCATTGGTTGACTTTCC |
|  | NtFLSF | CTTGAAGGGAAAAGGGGTTGG |
|  | NtFLSR | CGCAACTTCTCGCAGCCTCT |
|  | NtANSF | GTGCCTGGGTTACAACTTTTCTATG |
|  | NtANSR | CATTGCTTAGGATTTCAAGGGTGTC |
|  | NtEF1αF | TGGTTGTGACTTTTGGTCCCA |
|  | NtEF1αR | ACAAACCCACGCTTGAGATCC |
| *Dianthus caryophyllus* | DcFLSF | CGAGACCACACCGACATCAACA |
|  | DcFLSR | ACCGATAAAAACAGCAAACGAGG |
|  | DcDFRF | GGTTTCATTGGTTCATTGCTCAT |
|  | DcDFRR | CTTCTTCGTGTAGGTCCGCTTTC |
|  | DcGAPDHF | CACTCCATCACAGCCACACAAAAG |
|  | DcGAPDHR | CACGGAAAGCCATACCAGTCAAC |
| *Camellia japonica* | CjFLSF | AGTGTATGCCGAGAGGTTGCGT |
|  | CjFLSR | GAGCCCTGGGACTTCATTTGG |
|  | CjDFRF | CAATAAGAAGAAGATGACTGGCTGGAT |
|  | CjDFRR | GAGCGGTGATTAGGCTTGGTGG |
|  | Cj18SF | GACTCAACACGGGGAAACTTACC |
|  | Cj18SR | CAGACAAATCGCTCCACCAAC |
| *Rosa multifloral* | RmFLS1F | TGGAGGGATACGGAACATTTTTA |
|  | RmFLS1R | CACCACCTTGTGTAGATTCTTTGC |
|  | RmDFRF | CAAGGGCATTGAGGAGAACTTGC |
|  | RmDFRR | CCTGTGACTTTGACACGGACGA |
|  | RmGAPDHF | TGAAGGGTGGTGCCAAGAA |
|  | RmGAPDHR | AAGGGGAGCAAGACAGTTGG |
